# The Controversies Surrounding Acne and Suicide: Essential Knowledge for Clinicians

**DOI:** 10.7759/cureus.43867

**Published:** 2023-08-21

**Authors:** Nihit Gupta, Mayank Gupta

**Affiliations:** 1 Psychiatry, Dayton Children’s Hospital, Dayton, USA; 2 Psychiatry and Behavioral Sciences, Southwood Psychiatric Hospital, Pittsburgh, USA

**Keywords:** isotretinoin adverse effects, depression prevention, suicide risk, topical acne treatment, acne vulgaris. epidemiology. life style

## Abstract

Over the years, there have been numerous studies linking acne to depression and suicidal thoughts. Although the exact relationship between the two is still unclear, the stigma associated with acne can have a significant impact on an individual’s mental health. The critical appraisal of empirical evidence reveals a lack of understanding of the relationship between acne and suicide. Several studies have demonstrated an association between acne and suicide without causal links. Thus, it is clinically important to review the risks associated with isotretinoin and obtain informed consent regarding potential harm. The use of isotretinoin should be limited to cases of severe acne. There have been reports of an increased risk of suicide six months after the completion of isotretinoin treatment, so it is recommended that follow-up monitoring continue for up to one year following the completion of the treatment.

## Editorial

Despite the advances in cutting-edge empirical research, the trends in suicide deaths have perplexed the scientific community over the last several decades, as mortality related to medical reasons has plummeted in children and adolescents. According to the Center for Disease Control (CDC)'s National Center for Health Statistics (NCHS) data briefs, the suicide rate between the ages of 10 and 24 is currently estimated at 10.6 per 100,000 individuals, with a significant 56% increase between 2007 and 2017 [1]. In this highly funded and extensively researched field, the weak statistical association between acne and suicide has puzzled researchers, clinicians, and the public at large.

After its Food and Drug Administration (FDA) approval in 1982, isotretinoin was controversial for its teratogenic effects until it was linked to mental health conditions [2]. These links between suicide and acne date back 30 years, when an original French paper entitled "Isotretinoin and Depression: Let's Be Vigilant" raised safety concerns about the administration of isotretinoin to people with mental disorders [3]. This was concerning given that acne is a relatively common clinical condition affecting 9.4% of the global population, with post-pubertal boys being the most affected and also having severe forms of acne [4]. About 85% of people in the U.S. between the ages of 12 and 24 experience at least mild acne at some point in their lives [5].

At the end of the 20th century, there were many reports of the association between isotretinoin treatment of acne and mental disorders. These findings are being investigated globally by regulatory agencies. In the United States, the FDA issued a black box warning with isotretinoin due to reports of depression, suicide, and psychosis in 2005 [6]. However, in 2010, an influential and methodologically strong retrospective cohort study of 5756 Swedish individuals found an association between severe acne and the risk of suicide attempts. Its findings suggested that the risk of suicide significantly increased several years prior to the initiation of treatment with isotretinoin, and the suicide risk was heightened for a period of up to six months after treatment with isotretinoin was completed. However, no additional risk could be attributed to isotretinoin treatment [7]. The authors of the study recommended continued clinical monitoring for up to a year after treatment completion.

Another observation from a large population-based study reported a significant association between mental health symptoms, suicidal ideation, and substantial acne (odds ratio of 1.80, 95% confidence interval 1.30-2.50) [8]. The authors underscore that the psychiatric adverse effects associated with acne treatment may reflect the burden of substantial acne rather than an adverse reaction to the medication.

Given these controversies in the last two decades, there are reviews designed to understand these links. A meta-analysis conducted in 2021 that excluded studies treated with isotretinoin found that acne was positively associated with suicide (odds ratio [OR] 1.50, 95% confidence interval [95% CI]: 1.09-2.06, P = 0.004, I2 = 74.1%) [9]. Another review of 13 studies has found that low self-esteem is associated with acne vulgaris patients with a strong female preponderance. A minority of these patients seeking any form of treatment due to the severity of acne, associated bullying, and increased risk for clinical depression warrant earlier interventions [10].

Table 1 provides a summary of these rare and serious outcomes that have inspired more than two decades of scientific inquiries in chronological order about the association between acne, isotretinoin treatment, and suicide.

**Table 1:** Summary table of important articles reviewed

Important studies (in chronological order)	Key findings
Jick et al. [22]	A large population-based cohort study found no evidence of association between isotretinoin use and psychiatric disorders.
Ragmanauskaite et al. [23]	Acne can be more challenging in sexual and gender minority adolescents and requires specific psychosocial considerations.
Hull and D'Arcy [24]	When treating individuals with isotretinoin, it is paramount to inform patients and families about the risks of mental disorders and seek a psychiatric assessment.
Sundström et al. [7]	Increased risk of suicide attempts was observed six months after treatment with isotretinoin. Therefore, follow-up monitoring of behaviors for up to a year after completion of isotretinoin treatment were recommended.
Halvorsen et al. [8]	Adverse events of acne treatment including depression and suicidal ideations may reflect the burden of substantial acne and not the effects of medication.
Yang et al. [25]	Found in a large community sample that depression was more common in individuals with acne (0.77% compared to 0.56% in controls with P<0.0001).
Huang and Cheng [26]	Found no association between isotretinoin treatment and depression and concluded that acne treatment helped with depressive symptoms (RR 0.588, 95% CI: 0.382-0.904)
Prabhakar et al. [27]	Found no evidence of increased risk of death by suicide in individuals with dermatological conditions.
Xu et al. [9]	Metanalysis included 5 studies and 52,075 participants found significantly increase suicide risk associated with acne (OR 1.50, 95% CI: 1.09–2.06, P = 0.004, I2 = 74.1%). Recommended screening during active treatment.
Paljarvi et al. [15]	Isotretinoin independently was not associated with excess neuropsychiatric outcomes with odds ratio 0.8 (95% CI: 0.74-0.87).
Hefez et al. [28]	Multicenter prospective study in the implementation of Adolescent Depression Rating Scale (ADRS) for monitoring of depression symptoms before and during treatment with isotretinoin.

In the past three decades, isotretinoin, an active form of vitamin A, has been an effective treatment for acne since the 1980s. This drug is highly teratogenic, and it is recommended that females engage in at least two forms of contraception before commencing treatment with isotretinoin [11]. Isotretinoin has also been reported to cause neuropsychiatric side effects like depression, anxiety, suicidality, and psychosis [12].

Amidst these developments, in 2014, the UK’s Medicines and Healthcare Products Regulatory Agency (MHRA) concluded that the data were "insufficient to establish a causal association but could not rule out an association between isotretinoin and psychiatric disorders" [13]. In 2018, the European Medicines Agency (EMA) echoed similar concerns and issued a warning recommending reviewing the empirical data and integrating it into clinical practice to enhance safety and risk management [14].

Although a recent retrospective cohort study by Paljarvi et al. found that isotretinoin independently was not associated with excess neuropsychiatric outcomes with an odds ratio of 0.8 (95% confidence interval [CI] 0.74-0.87) [15], they found individuals with acne vulgaris have higher odds (1:46) of mental disorders compared to the matched group without acne. However, the risks were reduced with the isotretinoin treatment as compared to individuals treated with antibiotics. These findings underscore the effectiveness of isotretinoin in the treatment-resistant form of severe acne vulgaris, which attributes to the reduction in risks [15].

These findings were also replicated in other studies, and Ugonabo et al. found suicidal behavior during isotretinoin treatment was lower (0.10%; P = 0.082) than in the year prior to isotretinoin treatment (0.22%) and in the year following treatment (0.34%; P = 0.004) [16]. Another study reviewed the US FDA Adverse Event Reporting System from 1997 through 2017, enrolled in the iPLEDGE program [17]. Interestingly, deaths by suicide in patients on isotretinoin were found to be lower than those in the general US population [14].

In 2019, MHRA’s yellow card reporting scheme recorded 12 deaths associated with Roaccutane (isotretinoin) prescriptions, and out of them, 10 were suicides [18]. As a result, MHRA has reopened another investigation and, in April 2023, published a report from the Commission on Human Medicines (CHM) Isotretinoin Expert Working Group. The group recommended changing the product information on isotretinoin’s frequency of psychiatric side effects from ‘insufficient to establish an association’ to ‘not known’ [19]. They also concluded that the benefits outweigh the risks for severe acne, given that untreated severe acne is a risk for scarring, which has long-term psychological effects.

According to an expert review, the lack of randomized controlled trials (RCT) data as evidence of isotretinoin-associated psychiatric side effects is concluded to be "inadequate evidence." However, RCTs are not appropriate when studying rare outcomes [12], and therefore, warnings from case reports and database studies are good enough evidence to substantiate an association between treatment with isotretinoin and depression, suicidal thoughts, and behaviors [20].

Several case reports and case series indicate an increased risk of neuropsychiatric side effects, and there is evidence that there may be a dose-response effect, a temporal association, and a possible causal relationship between isotretinoin and psychiatric adverse effects. As a result, guidelines are very strict about using isotretinoin for severe acne and support warning patients about isotretinoin's potential for neuropsychiatric side effects [12].

There is a lack of clarity about the relationship between acne and suicide. Though several studies have demonstrated an association between the two, there is no evidence of any causal links. For severe acne, isotretinoin remains one of the most prescribed medications, and according to the DrugStats database, there were an estimated 1,572,313 prescriptions of isotretinoin in an estimated 419,799 patients in the United States in the year 2020 [21]. The MRHA expert safety review appropriately summarizes key recommendations regarding the use of isotretinoin in any individual, such as the need for better information to be provided to families, regular monitoring for side effects, the use of isotretinoin, which should be reserved for severe acne, and lastly, the need for better communication about the risk associated with the use [19].

Integrating evidence in clinical practice

It is recommended that the prescribing physician obtain informed consent from patients and their families about the potential risks of mental disorders associated with the use of isotretinoin and request psychiatric evaluations when necessary. It is necessary to educate both professionals and the general population and collaborate with dermatologists about the evidence of risk. It is imperative to enroll in the FDA's iPLEDGE REMS safety program to manage the risk of isotretinoin’s teratogenicity and minimize fetal exposure. Before starting isotretinoin treatment for acne, screening for mental disorders is recommended. Topical isotretinoin over systemic administration should be favored as much as possible to minimize side effects. These clinical considerations may help improve long-term outcomes and enhance patient safety in real-world clinical practice. With reports of increased suicide risk six months post-completion of treatment with isotretinoin, continued follow-up monitoring for up to one year is recommended.
